# Supplementary material for: Effect of Tertiary Lymphoid Structures on Prognosis of Patients with Hepatocellular Carcinoma and Preliminary Exploration of Its Formation Mechanism
Source: Cancers (Basel). 2022 Oct 21;14(20):5157. doi: 10.3390/cancers14205157 (PMC9601110; doi:10.3390/cancers14205157)
Supplement: Supplementary file 1 [file cancers-14-05157-s001.zip › cancers-1942510-supplementary.pdf]

supplementary materials

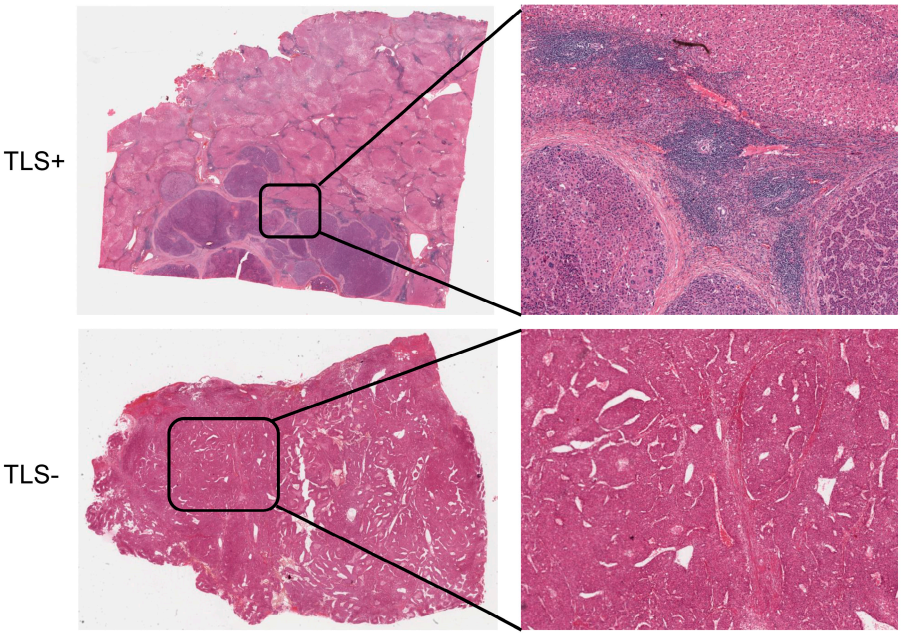

Figure S1: According to the HE staining results in TCGA data, the patients were divided into the TLS+ (n=195) group and TLS- (136) group.

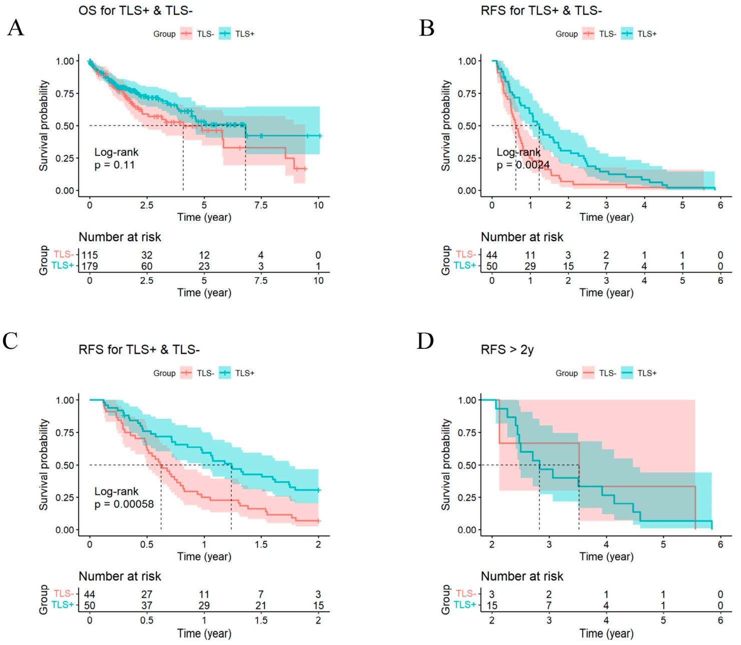

Figure S2: TCGA data, effects of TLSs on overall survival and recurrence-free survival in patients with hepatocellular carcinoma: (A) overall survival; (B) recurrence-free survival; (C) early recurrence-free survival and (D) late recurrence-free survival.

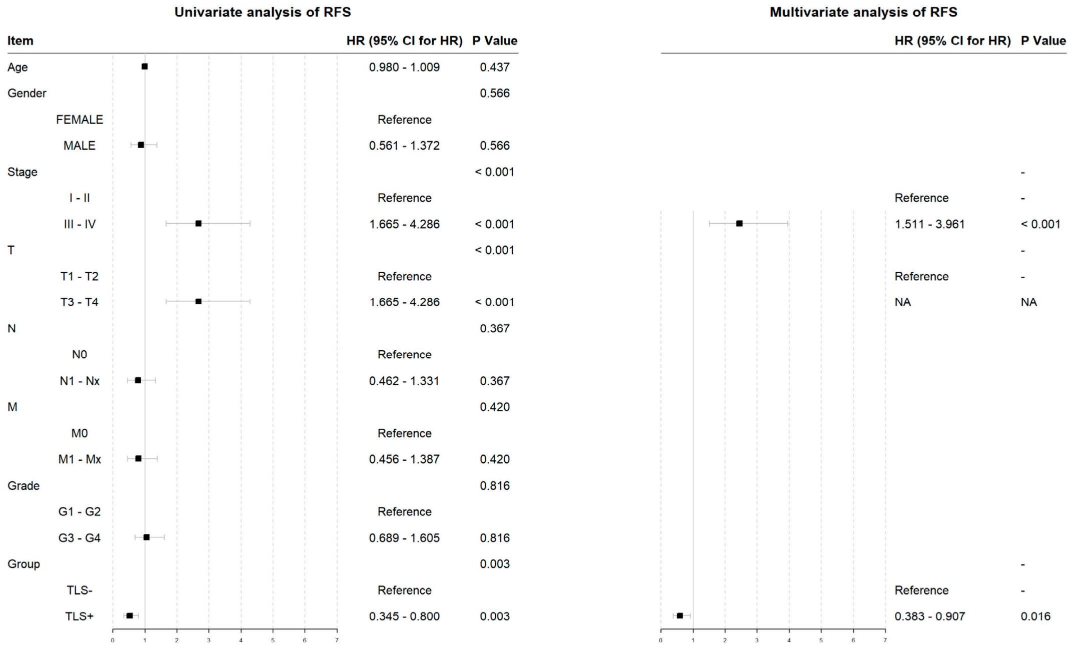

Figure S3: Univariate and multivariate Cox regression analyses of risk factors associated with recurrence-free survival from TCGA data.

Table S1: According to the HE staining results, HCC patients were divided into TLS+ group and TLS- group according to the presence or absence of TLS, which were obtained from TCGA data

ID	Type	TLS+/-
TCGA-2V-A95S-01A	YES	TLS+
TCGA-2Y-A9GS-01A	YES	TLS+
TCGA-2Y-A9GT-01A	YES	TLS+
TCGA-2Y-A9GU-01A	YES	TLS+
TCGA-2Y-A9GW-01A	YES	TLS+
TCGA-2Y-A9GX-01A	YES	TLS+
TCGA-2Y-A9GY-01A	YES	TLS+
TCGA-2Y-A9H0-01A	YES	TLS+
TCGA-2Y-A9H1-01A	YES	TLS+
TCGA-2Y-A9H2-01A	YES	TLS+
TCGA-2Y-A9H3-01A	YES	TLS+
TCGA-2Y-A9H4-01A	YES	TLS+
TCGA-2Y-A9H5-01A	YES	TLS+
TCGA-2Y-A9H6-01A	YES	TLS+
TCGA-2Y-A9H7-01A	YES	TLS+
TCGA-2Y-A9HA-01A	YES	TLS+
TCGA-2Y-A9HB-01A	None	TLS-
TCGA-3K-AAZ8-01A	None	TLS-
TCGA-4R-AA8I-01A	None	TLS-

TCGA-5C-A9VG-01A	None	TLS-
TCGA-5C-A9VH-01A	None	TLS-
TCGA-5C-AAPD-01A	YES	TLS+
TCGA-5R-AA1C-01A	None	TLS-
TCGA-5R-AA1D-01A	None	TLS-
TCGA-5R-AAAM-01A	None	TLS-
TCGA-BC-4072-01B	None	TLS-
TCGA-BC-A10S-01A	YES	TLS+
TCGA-BC-A10T-01A	None	TLS-
TCGA-BC-A10U-01A	None	TLS-
TCGA-BC-A10X-01A	None	TLS-
TCGA-BC-A10Y-01A	None	TLS-
TCGA-BC-A10Z-01A	None	TLS-
TCGA-BC-A110-01A	None	TLS-
TCGA-BC-A112-01A	YES	TLS+
TCGA-BC-A216-01A	YES	TLS+
TCGA-BC-A217-01A	YES	TLS+
TCGA-BC-A3KF-01A	None	TLS-
TCGA-BC-A3KG-01A	None	TLS-
TCGA-BC-A5W4-01A	None	TLS-
TCGA-BC-A69H-01A	None	TLS-
TCGA-BC-A69I-01A	None	TLS-
TCGA-BD-A2L6-01A	None	TLS-
TCGA-BD-A3ER-01A	None	TLS-
TCGA-BW-A5NO-01A	YES	TLS+
TCGA-BW-A5NQ-01A	YES	TLS+
TCGA-CC-5258-01A	YES	TLS+
TCGA-CC-5259-01A	None	TLS-
TCGA-CC-5260-01A	YES	TLS+
TCGA-CC-5261-01A	YES	TLS+
TCGA-CC-5262-01A	None	TLS-
TCGA-CC-5263-01A	None	TLS-
TCGA-CC-5264-01A	None	TLS-
TCGA-CC-A123-01A	None	TLS-
TCGA-CC-A1HT-01A	YES	TLS+
TCGA-CC-A3M9-01A	None	TLS-
TCGA-CC-A3MA-01A	YES	TLS+
TCGA-CC-A3MB-01A	None	TLS-
TCGA-CC-A3MC-01A	None	TLS-
TCGA-CC-A5UC-01A	YES	TLS+
TCGA-CC-A5UD-01A	None	TLS-
TCGA-CC-A5UE-01A	None	TLS-
TCGA-CC-A7IE-01A	YES	TLS+

TCGA-CC-A7IF-01A	None	TLS-
TCGA-CC-A7IG-01A	YES	TLS+
TCGA-CC-A7IH-01A	None	TLS-
TCGA-CC-A7II-01A	None	TLS-
TCGA-CC-A7IJ-01A	None	TLS-
TCGA-CC-A7IK-01A	None	TLS-
TCGA-CC-A7IL-01A	None	TLS-
TCGA-CC-A8HS-01A	None	TLS-
TCGA-CC-A8HT-01A	None	TLS-
TCGA-CC-A8HU-01A	None	TLS-
TCGA-CC-A9FS-01A	YES	TLS+
TCGA-CC-A9FU-01A	None	TLS-
TCGA-CC-A9FV-01A	None	TLS-
TCGA-CC-A9FW-01A	YES	TLS+
TCGA-DD-A113-01A	YES	TLS+
TCGA-DD-A114-01A	YES	TLS+
TCGA-DD-A116-01A	YES	TLS+
TCGA-DD-A118-01A	None	TLS-
TCGA-DD-A119-01A	YES	TLS+
TCGA-DD-A11A-01A	None	TLS-
TCGA-DD-A11B-01A	YES	TLS+
TCGA-DD-A11C-01A	None	TLS-
TCGA-DD-A1EA-01A	YES	TLS+
TCGA-DD-A1EB-01A	None	TLS-
TCGA-DD-A1ED-01A	YES	TLS+
TCGA-DD-A1EE-01A	None	TLS-
TCGA-DD-A1EF-01A	YES	TLS+
TCGA-DD-A1EI-01A	YES	TLS+
TCGA-DD-A1EL-01A	YES	TLS+
TCGA-DD-A39V-01A	YES	TLS+
TCGA-DD-A39W-01A	None	TLS-
TCGA-DD-A39X-01A	YES	TLS+
TCGA-DD-A39Y-01A	YES	TLS+
TCGA-DD-A39Z-01A	None	TLS-
TCGA-DD-A3A1-01A	YES	TLS+
TCGA-DD-A3A2-01A	None	TLS-
TCGA-DD-A3A3-01A	None	TLS-
TCGA-DD-A3A5-01A	None	TLS-
TCGA-DD-A3A6-01A	None	TLS-
TCGA-DD-A3A8-01A	None	TLS-
TCGA-DD-A3A9-01A	None	TLS-
TCGA-DD-A4NA-01A	YES	TLS+
TCGA-DD-A4NB-01A	YES	TLS+

TCGA-DD-A4NF-01A	YES	TLS+
TCGA-DD-A4NG-01A	YES	TLS+
TCGA-DD-A4NI-01A	YES	TLS+
TCGA-DD-A4NJ-01A	YES	TLS+
TCGA-DD-A4NK-01A	None	TLS-
TCGA-DD-A4NL-01A	None	TLS-
TCGA-DD-A4NN-01A	YES	TLS+
TCGA-DD-A4NO-01A	YES	TLS+
TCGA-DD-A4NP-01A	None	TLS-
TCGA-DD-A4NR-01A	YES	TLS+
TCGA-DD-A4NV-01A	None	TLS-
TCGA-DD-A73A-01A	YES	TLS+
TCGA-DD-A73B-01A	YES	TLS+
TCGA-DD-A73C-01A	None	TLS-
TCGA-DD-A73D-01A	YES	TLS+
TCGA-DD-A73E-01A	YES	TLS+
TCGA-DD-A73F-01A	YES	TLS+
TCGA-DD-A73G-01A	YES	TLS+
TCGA-DD-AA3A-01A	None	TLS-
TCGA-DD-AAC8-01A	YES	TLS+
TCGA-DD-AAC9-01A	YES	TLS+
TCGA-DD-AACA-01A	YES	TLS+
TCGA-DD-AACB-01A	YES	TLS+
TCGA-DD-AACC-01A	YES	TLS+
TCGA-DD-AACD-01A	YES	TLS+
TCGA-DD-AACE-01A	YES	TLS+
TCGA-DD-AACF-01A	None	TLS-
TCGA-DD-AACG-01A	None	TLS-
TCGA-DD-AACH-01A	YES	TLS+
TCGA-DD-AACI-01A	YES	TLS+
TCGA-DD-AACJ-01A	None	TLS-
TCGA-DD-AACK-01A	None	TLS-
TCGA-DD-AACL-01A	None	TLS-
TCGA-DD-AACN-01A	None	TLS-
TCGA-DD-AACO-01A	None	TLS-
TCGA-DD-AACP-01A	YES	TLS+
TCGA-DD-AACQ-01A	None	TLS-
TCGA-DD-AACS-01A	None	TLS-
TCGA-DD-AACT-01A	YES	TLS+
TCGA-DD-AACU-01A	YES	TLS+
TCGA-DD-AACV-01A	None	TLS-
TCGA-DD-AACW-01A	None	TLS-
TCGA-DD-AACX-01A	None	TLS-

TCGA-DD-AACY-01A	None	TLS-
TCGA-DD-AACZ-01A	None	TLS-
TCGA-DD-AAD0-01A	None	TLS-
TCGA-DD-AAD1-01A	YES	TLS+
TCGA-DD-AAD2-01A	YES	TLS+
TCGA-DD-AAD3-01A	YES	TLS+
TCGA-DD-AAD5-01A	None	TLS-
TCGA-DD-AAD6-01A	None	TLS-
TCGA-DD-AAD8-01A	None	TLS-
TCGA-DD-AADA-01A	YES	TLS+
TCGA-DD-AADB-01A	YES	TLS+
TCGA-DD-AADC-01A	YES	TLS+
TCGA-DD-AADD-01A	None	TLS-
TCGA-DD-AADF-01A	None	TLS-
TCGA-DD-AADG-01A	None	TLS-
TCGA-DD-AADI-01A	YES	TLS+
TCGA-DD-AADJ-01A	None	TLS-
TCGA-DD-AADK-01A	YES	TLS+
TCGA-DD-AADL-01A	None	TLS-
TCGA-DD-AADM-01A	YES	TLS+
TCGA-DD-AADN-01A	YES	TLS+
TCGA-DD-AADO-01A	YES	TLS+
TCGA-DD-AADP-01A	YES	TLS+
TCGA-DD-AADQ-01A	YES	TLS+
TCGA-DD-AADR-01A	None	TLS-
TCGA-DD-AADS-01A	YES	TLS+
TCGA-DD-AADU-01A	None	TLS-
TCGA-DD-AADV-01A	None	TLS-
TCGA-DD-AADW-01A	YES	TLS+
TCGA-DD-AADY-01A	None	TLS-
TCGA-DD-AAE0-01A	YES	TLS+
TCGA-DD-AAE1-01A	YES	TLS+
TCGA-DD-AAE2-01A	YES	TLS+
TCGA-DD-AAE3-01A	None	TLS-
TCGA-DD-AAE4-01A	None	TLS-
TCGA-DD-AAE6-01A	None	TLS-
TCGA-DD-AAE7-01A	YES	TLS+
TCGA-DD-AAE9-01A	YES	TLS+
TCGA-DD-AAEA-01A	None	TLS-
TCGA-DD-AAEB-01A	None	TLS-
TCGA-DD-AAED-01A	None	TLS-
TCGA-DD-AAEE-01A	None	TLS-
TCGA-DD-AAEG-01A	YES	TLS+

TCGA-DD-AAEH-01A	None	TLS-
TCGA-DD-AAEI-01A	None	TLS-
TCGA-DD-AAEK-01A	YES	TLS+
TCGA-DD-AAVP-01A	YES	TLS+
TCGA-DD-AAVQ-01A	YES	TLS+
TCGA-DD-AAVR-01A	YES	TLS+
TCGA-DD-AAVS-01A	YES	TLS+
TCGA-DD-AAVU-01A	YES	TLS+
TCGA-DD-AAVV-01A	YES	TLS+
TCGA-DD-AAVW-01A	YES	TLS+
TCGA-DD-AAVX-01A	YES	TLS+
TCGA-DD-AAVY-01A	YES	TLS+
TCGA-DD-AAVZ-01A	None	TLS-
TCGA-DD-AAW0-01A	YES	TLS+
TCGA-DD-AAW1-01A	YES	TLS+
TCGA-DD-AAW2-01A	YES	TLS+
TCGA-DD-AAW3-01A	YES	TLS+
TCGA-ED-A4XI-01A	YES	TLS+
TCGA-ED-A5KG-01A	YES	TLS+
TCGA-ED-A627-01A	None	TLS-
TCGA-ED-A66X-01A	YES	TLS+
TCGA-ED-A66Y-01A	YES	TLS+
TCGA-ED-A7PX-01A	None	TLS-
TCGA-ED-A7PY-01A	None	TLS-
TCGA-ED-A7PZ-01A	None	TLS-
TCGA-ED-A7XO-01A	YES	TLS+
TCGA-ED-A7XP-01A	None	TLS-
TCGA-ED-A82E-01A	YES	TLS+
TCGA-ED-A8O5-01A	YES	TLS+
TCGA-ED-A8O6-01A	YES	TLS+
TCGA-ED-A97K-01A	YES	TLS+
TCGA-EP-A12J-01A	None	TLS-
TCGA-EP-A26S-01A	None	TLS-
TCGA-EP-A2KB-01A	YES	TLS+
TCGA-EP-A2KC-01A	None	TLS-
TCGA-EP-A3JL-01A	YES	TLS+
TCGA-EP-A3RK-01A	YES	TLS+
TCGA-ES-A2HS-01A	None	TLS-
TCGA-ES-A2HT-01A	None	TLS-
TCGA-FV-A23B-01A	YES	TLS+
TCGA-FV-A2QQ-01A	None	TLS-
TCGA-FV-A2QR-01A	None	TLS-
TCGA-FV-A3I1-01A	YES	TLS+

TCGA-FV-A3R3-01A	None	TLS-
TCGA-FV-A495-01A	YES	TLS+
TCGA-FV-A496-01A	None	TLS-
TCGA-FV-A4ZP-01A	YES	TLS+
TCGA-FV-A4ZQ-01A	YES	TLS+
TCGA-G3-A25T-01A	YES	TLS+
TCGA-G3-A25U-01A	None	TLS-
TCGA-G3-A25V-01A	YES	TLS+
TCGA-G3-A25X-01A	YES	TLS+
TCGA-G3-A25Y-01A	YES	TLS+
TCGA-G3-A25Z-01A	YES	TLS+
TCGA-G3-A3CI-01A	YES	TLS+
TCGA-G3-A3CK-01A	YES	TLS+
TCGA-G3-A5SJ-01A	YES	TLS+
TCGA-G3-A5SK-01A	YES	TLS+
TCGA-G3-A5SL-01A	YES	TLS+
TCGA-G3-A5SM-01A	YES	TLS+
TCGA-G3-A6UC-01A	YES	TLS+
TCGA-G3-A7M5-01A	YES	TLS+
TCGA-G3-A7M6-01A	YES	TLS+
TCGA-G3-A7M7-01A	YES	TLS+
TCGA-G3-A7M8-01A	YES	TLS+
TCGA-G3-A7M9-01A	None	TLS-
TCGA-G3-AAUZ-01A	YES	TLS+
TCGA-G3-AAV0-01A	None	TLS-
TCGA-G3-AAV1-01A	YES	TLS+
TCGA-G3-AAV2-01A	YES	TLS+
TCGA-G3-AAV3-01A	YES	TLS+
TCGA-G3-AAV4-01A	YES	TLS+
TCGA-G3-AAV6-01A	YES	TLS+
TCGA-G3-AAV7-01A	YES	TLS+
TCGA-GJ-A3OU-01A	YES	TLS+
TCGA-GJ-A6C0-01A	None	TLS-
TCGA-GJ-A9DB-01A	None	TLS-
TCGA-HP-A5MZ-01A	YES	TLS+
TCGA-HP-A5N0-01A	None	TLS-
TCGA-K7-A5RF-01A	None	TLS-
TCGA-K7-A5RG-01A	YES	TLS+
TCGA-K7-A6G5-01A	YES	TLS+
TCGA-K7-AAU7-01A	YES	TLS+
TCGA-KR-A7K0-01A	None	TLS-
TCGA-KR-A7K2-01A	None	TLS-
TCGA-KR-A7K7-01A	None	TLS-

TCGA-KR-A7K8-01A	None	TLS-
TCGA-LG-A6GG-01A	YES	TLS+
TCGA-LG-A9QC-01A	YES	TLS+
TCGA-MI-A75C-01A	YES	TLS+
TCGA-MI-A75E-01A	YES	TLS+
TCGA-MI-A75G-01A	YES	TLS+
TCGA-MI-A75H-01A	YES	TLS+
TCGA-MI-A75I-01A	YES	TLS+
TCGA-MR-A520-01A	None	TLS-
TCGA-MR-A8JO-01A	YES	TLS+
TCGA-NI-A4U2-01A	None	TLS-
TCGA-NI-A8LF-01A	YES	TLS+
TCGA-O8-A75V-01A	YES	TLS+
TCGA-PD-A5DF-01A	None	TLS-
TCGA-QA-A7B7-01A	YES	TLS+
TCGA-RC-A6M3-01A	None	TLS-
TCGA-RC-A6M4-01A	YES	TLS+
TCGA-RC-A6M5-01A	YES	TLS+
TCGA-RC-A6M6-01A	YES	TLS+
TCGA-RC-A7S9-01A	YES	TLS+
TCGA-RC-A7SB-01A	None	TLS-
TCGA-RC-A7SF-01A	YES	TLS+
TCGA-RC-A7SH-01A	YES	TLS+
TCGA-RC-A7SK-01A	YES	TLS+
TCGA-RG-A7D4-01A	YES	TLS+
TCGA-T1-A6J8-01A	YES	TLS+
TCGA-UB-A7MC-01A	YES	TLS+
TCGA-UB-A7MD-01A	YES	TLS+
TCGA-UB-A7ME-01A	YES	TLS+
TCGA-UB-A7MF-01A	YES	TLS+
TCGA-UB-AA0U-01A	YES	TLS+
TCGA-UB-AA0V-01A	None	TLS-
TCGA-WJ-A86L-01A	YES	TLS+
TCGA-WQ-A9G7-01A	YES	TLS+
TCGA-WQ-AB4B-01A	YES	TLS+
TCGA-WX-AA46-01A	None	TLS-
TCGA-WX-AA47-01A	None	TLS-
TCGA-XR-A8TC-01A	None	TLS-
TCGA-XR-A8TD-01A	None	TLS-
TCGA-XR-A8TE-01A	YES	TLS+
TCGA-XR-A8TF-01A	None	TLS-
TCGA-XR-A8TG-01A	YES	TLS+
TCGA-YA-A8S7-01A	YES	TLS+

TCGA-ZP-A9CV-01A	YES	TLS+
TCGA-ZP-A9CY-01A	YES	TLS+
TCGA-ZP-A9CZ-01A	YES	TLS+
TCGA-ZP-A9D0-01A	None	TLS-
TCGA-ZP-A9D1-01A	YES	TLS+
TCGA-ZP-A9D2-01A	YES	TLS+
TCGA-ZP-A9D4-01A	YES	TLS+
TCGA-ZS-A9CD-01A	YES	TLS+
TCGA-ZS-A9CE-01A	YES	TLS+
TCGA-ZS-A9CF-01A	YES	TLS+
TCGA-ZS-A9CG-01A	YES	TLS+

Table S2. Clinicopathological characteristics of 150 HCC patients of HCC patients in Xijing Hospital.

**Characteristics**	**n**	**%**
TLSs		
Positive	61	40.7
Negative	89	59.3
Sex		
Male	125	83.3
Female	25	16.7
Age		
>60	46	30.7
≤60	104	69.3
ALT, μ/L		
>50	80	53.3
≤50	70	46.7
AST, μ/L		
>40	58	38.7
≤40	92	61.3
HBsAg		
positive	122	81.3
negative	28	18.7
TBil, μmol/L		
>14.3	44	29.3
≤14.3	106	70.7
AFP, ng/ml		
>7.79	56	37.3
≤7.79	94	62.7
Liver cirrhosis		
Yes	94	62.7
No	56	37.3
Lymph, 10E9/L		

>1.1	109	72.7
≤1.1	41	27.3
ALP, μ/L		
>125	45	30.0
≤125	105	70.0
ALB, g/L		
>40	112	74.7
≤40	38	25.3
Tumour number		
single	128	85.3
multiple	22	15.7
Tumour diameter, cm		
>7.71	67	44.7
≤7.71	83	55.3
Tumor capsule		
Yes	100	66.7
No	50	33.3
Cell differentiation		
poor/moderate	27	18.0
well	123	82.0
Portal vein invasion		
Yes	12	0.80
No	138	92.0
Cancer emboli		
Yes	91	60.7
No	59	39.3
Child grade		
A	146	97.3
B/C	4	0.70
AJCC Stage		
I+II	78	52.0
III+IV	72	48
BCLC		
B+C	65	76.5
0+A	85	23.5
